# Acute Decompensated Heart Failure in a Woman With Eisenmenger Syndrome: A Case Report

**DOI:** 10.7759/cureus.95399

**Published:** 2025-10-25

**Authors:** Ayobami B Omodara, Annabelle Milorde Attolico, Muneer Shoukath, Mita Kale

**Affiliations:** 1 Cardiology, Southend University Hospital NHS Foundation Trust, Essex, GBR; 2 Internal Medicine, Southend University Hospital NHS Foundation Trust, Essex, GBR; 3 Internal Medicine, Manchester University Hospitals NHS Foundation Trust, Manchester, GBR

**Keywords:** case report, complications of eisenmenger, congenital heart disease, congestive heart failure, eisenmenger, macitentan, pulmonary hypertension

## Abstract

Eisenmenger syndrome (ES) is a rare and serious complication of congenital heart disease (CHD), characterized by intracardiac shunting, pulmonary hypertension, reversal of blood flow, and cyanosis. Management primarily involves pharmacotherapy to improve survival and prevent complications, with surgical or interventional closure considered in early-stage cases.

We present a case of a 45-year-old woman, adopted at birth, who was diagnosed with ES around 10 years ago, which was confirmed through right heart catheterization, unbeknownst to the acute clinicians. She was admitted this time with features of acute decompensated heart failure. Her underlying condition, major aortopulmonary collateral arteries (MAPCA), had been intermittently monitored. Upon admission, she displayed classic heart failure symptoms, alongside elevated hematocrit and severely reduced but extremely well-tolerated oxygen saturation. Diagnostic evaluation, including echocardiography, confirmed pulmonary hypertension and right-to-left shunting, as the history was elusive to start with.

This case highlights the diagnostic and management challenges associated with ES, particularly in patients with limited medical history. It emphasizes the importance of maintaining a high index of suspicion in the presence of chronic hypoxia and abnormal hematologic parameters. We discuss the role of echocardiography and adjunctive imaging in diagnosis, review the pathophysiologic mechanisms underlying ES, and outline current therapeutic strategies, including the use of endothelin receptor antagonists in the management of pulmonary arterial hypertension (PAH).

## Introduction

Congenital heart diseases (CHDs) encompass a group of structural heart malformations present at birth, which can vary widely in their severity and impact on cardiac function [1]. These defects are commonly classified in several ways, including physiological distinctions such as cyanotic versus acyanotic conditions, anatomical categories like septal defects, outflow tract obstructions, great vessel abnormalities, and complex lesions, as well as clinical presentation-based categories, such as heart failure-dominant, cyanosis-dominant, or obstructive without shunt.

In contrast, inherited cardiac conditions (ICCs), which are distinct from CHDs, result from genetic mutations or abnormalities passed down through family lines. Examples of ICCs include hypertrophic obstructive cardiomyopathy (HOCM), dilated cardiomyopathy (DCM), arrhythmogenic ventricular cardiomyopathy (AVC), Brugada syndrome, and long QT syndrome (LQTS), among others.

Among congenital heart defects, the most prevalent subtypes include ventricular septal defect (VSD), atrial septal defect, patent ductus arteriosus, and tetralogy of Fallot. The severity of these defects is largely determined by the size of the abnormality and the extent of intracardiac communication, as seen in conditions like Eisenmenger syndrome (ES) [2]. Early identification of characteristic clinical signs, prompt diagnosis, and timely intervention are essential in preventing complications such as heart failure and other long-term sequelae. With appropriate management, the quality of life can be significantly improved through better exercise tolerance and a reduction in cardiopulmonary symptoms.

## Case presentation

Our patient is a 45-year-old lady who first saw her primary care physician on admission day, with symptoms including increased breathlessness, bilateral leg swelling, and reduced exercise tolerance worsened by exertion. She also had orthopnea and gained over 12 kilograms in a few months, without reporting palpitations or chest pain. The patient initially withheld her history of a major heart disease due to the fear of being blamed for defaulting on medical follow-up. As a result, her baseline labs and vitals were unknown.

Apart from heart failure, there was no other noteworthy medical history or family history of CHD. An ex-smoker with a 10-pack-year smoking history and who quit 20 years ago, she rarely drank alcohol, and her exercise tolerance had decreased to 45 meters from about 500 meters a year earlier. Of note, she had no known haematological malignancy. 

Physical examination showed oxygen saturation of 84%, heart rate of 112 beats per minute, and a mildly elevated jugular venous pulse. She had bi-basal inspiratory crackles and bilateral pitting lower limb oedema. Precordial examination showed hyperactivity and a right ventricular heave, heart sounds of 1 and 2 with P2 accentuation and a grade 4 pansystolic murmur, most pronounced around the left lower sternal border. This evidence supported a preliminary diagnosis of decompensated congestive cardiac failure (CCF) secondary to chronic valvular/structural heart disease.

**Figure 1:**
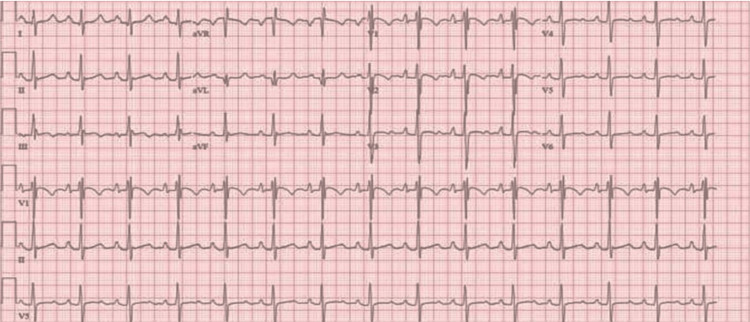
Abnormal electrocardiogram showing nonspecific changes suggesting right axis deviation, RsR' pattern in V1, and abnormal ventricular repolarization

In her initial blood tests, we observed polycythemia with haemoglobin levels at 185g/L (adult female range: 115-165g/L) and an elevated haematocrit value of 57% (0.57). On further testing, N-terminal pro-B-type natriuretic peptide (NT-proBNP) was 1,145 ng/L (normal: <125 ng/L). The electrocardiogram (ECG) showed nonspecific changes with abnormal ventricular repolarization (Figure 1).

**Figure 2:**
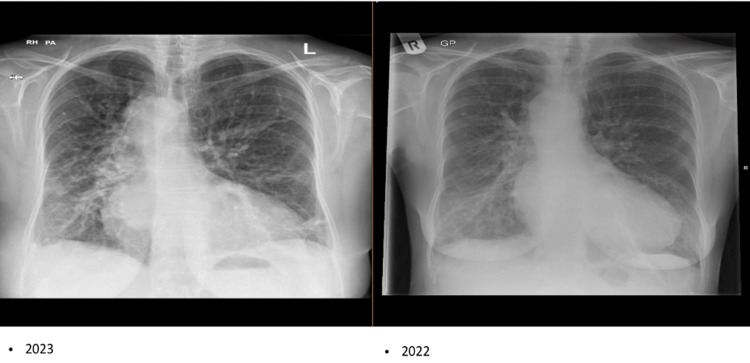
The chest X-ray only revealed some signs of fluid overload in the image on the left. Increased cardiothoracic ratio (CTR). Presence of Kerley b lines. Upper lobe diversion and right-sided pleural effusion with bluntness of costophrenic angles

Subsequently, a chest X-ray was done on admission (Figure 2, X-ray on the left) that showed signs of fluid overload, increased bronchial vascular markers, cardiomegaly, and pleural effusion. 

**Figure 3:**
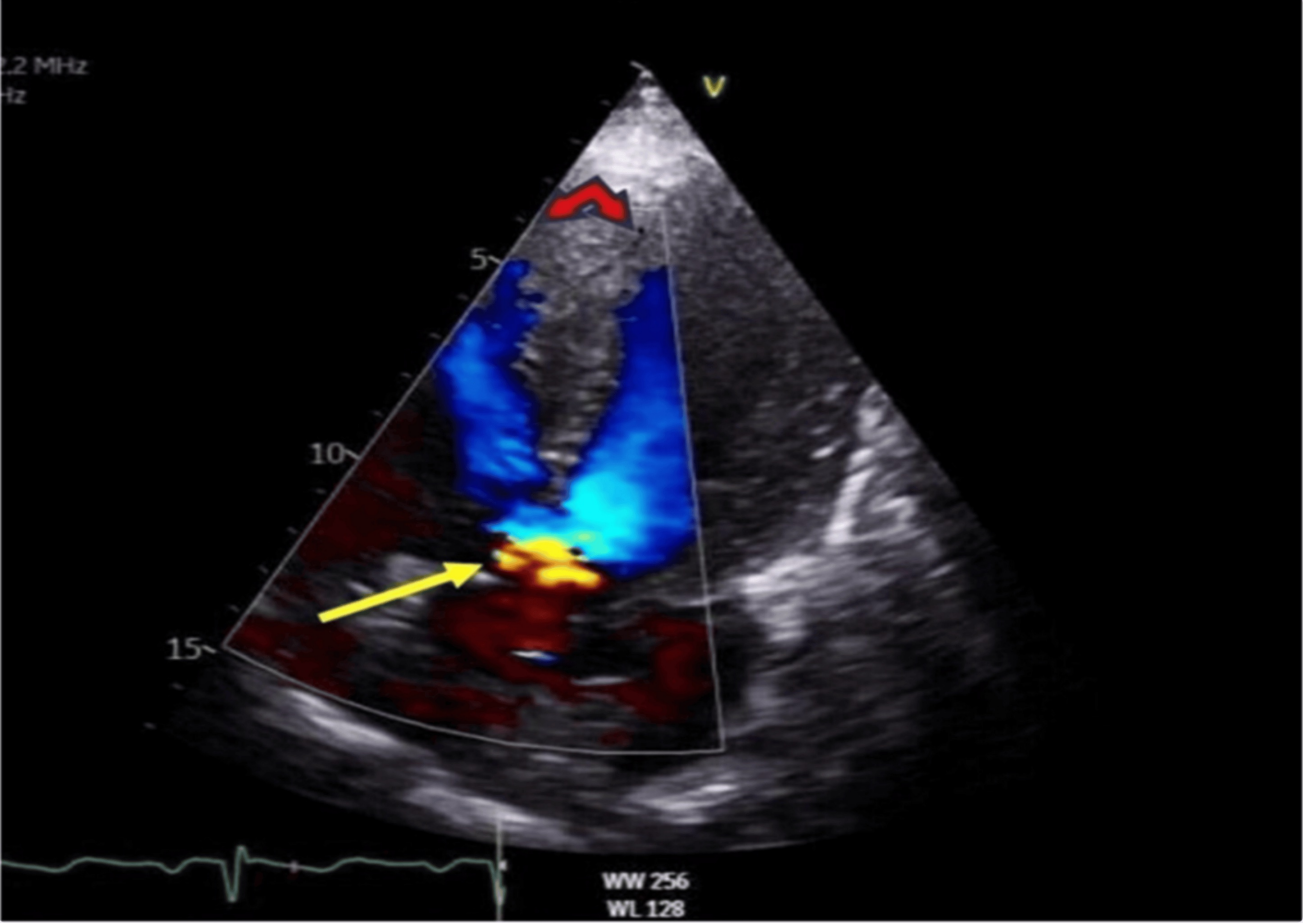
Yellow arrow, large VSD with bidirectional flow, a hallmark feature of Eisenmenger indicating significant right ventricular pressure. Red double arrow: backflow into both the left ventricle and right ventricle simultaneously. Doppler effect-apical four-chamber view VSD: ventricular septal defect; LV: left ventricle The left ventricle was normal in size and thickness. Additionally, the echocardiogram displayed an overriding aorta, shown in Figure 3. Echo revealed good systolic function and an LV ejection fraction of 55%

**Figure 4:**
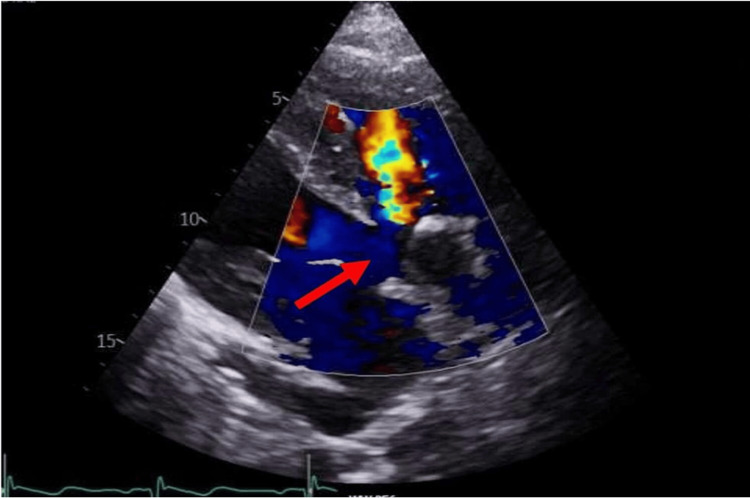
ECHO image in parasternal long axis view (PLAX) across the septal defect. Red arrow shows membranous ventricular (RV) septal defect with overriding aorta and moderate jet of aortic regurgitation directed to the right ventricular cavity Furthermore, moderate aortic regurgitation was noted. There was no indication of significant RV dilatation or hypertrophy. Despite established pulmonary hypertension, we couldn’t accurately estimate pulmonary artery systolic pressure (PASP) due to pulmonary atresia

**Figure 5:**
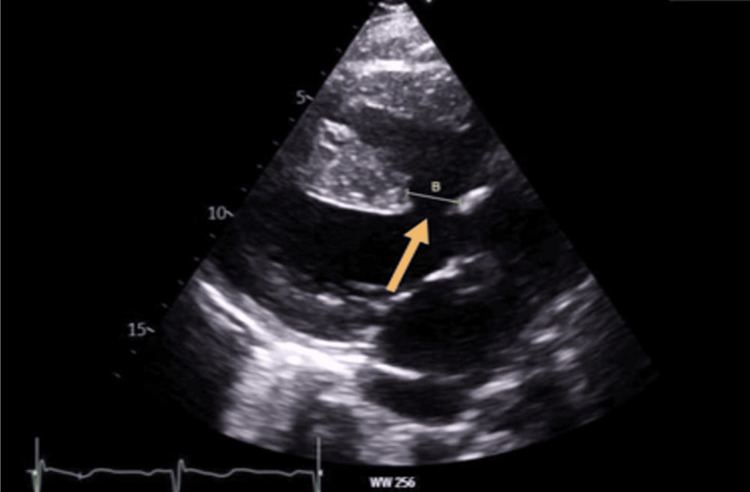
Yellow arrow depicts ventricular septal defect (VSD) measuring B=2cm in parasternal long axis ECHO view (PLAX). Yellow arrow shows similar finding in apical 4 chamber echocardiographic view

**Figure 6:**
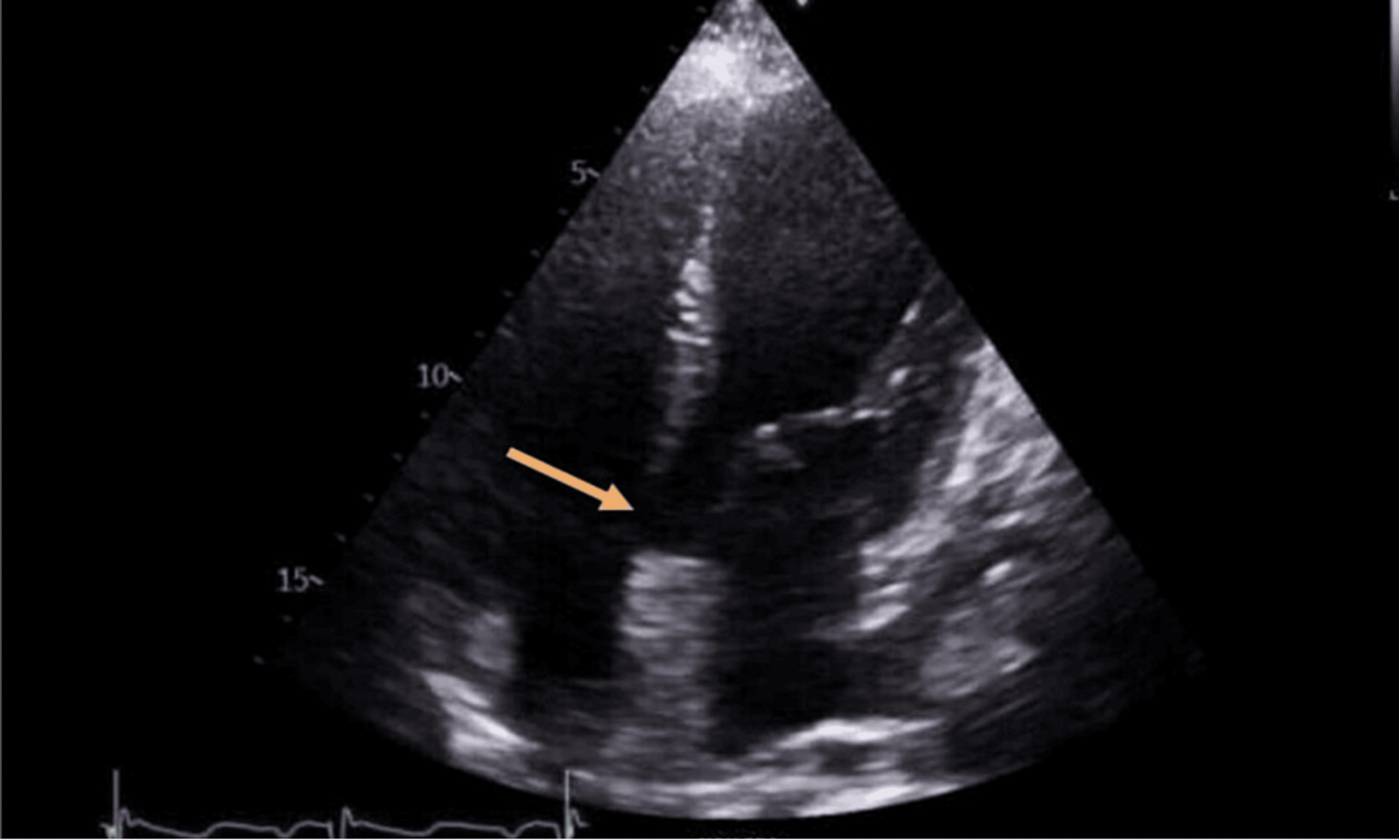
Yellow arrow shows a large membranous ventricular septal defect (VSD) in apical four-chamber echocardiographic view

The next morning echocardiogram was carried out, which showed a VSD with overriding aorta (Figures 3-6). 

Further review of her records revealed the patient was previously diagnosed with complex uncorrected CHD, including an uncorrected VSD, pulmonary atresia, and multiple aortopulmonary shunts (MAPACs).

Initially, IV furosemide and fluid restriction were planned, but due to the patient's extreme needle phobia, we administered furosemide 40 mg twice daily and then switched to bumetanide later on. She was discharged on oral bumetanide 2 mg OD. Despite optimal oxygen therapy via 24% Venturi, saturation ranged from 82% to 85%. CPAP initiation was considered but proved unnecessary.

During her admission to the cardiology unit, the patient demonstrated significant clinical improvement and achieved euvolemia. Given that she was previously on macitentan but reported noncompliance, we decided to offer her thorough counselling and resumed the medication. Oral macitentan (10 mg daily) was reinitiated following a negative pregnancy test and advised regarding its teratogenic risks. The case was reviewed in consultation with the adult congenital heart disease (ACHD) team, who advised targeting an oxygen saturation level of >83% in room air.

**Figure 7:**
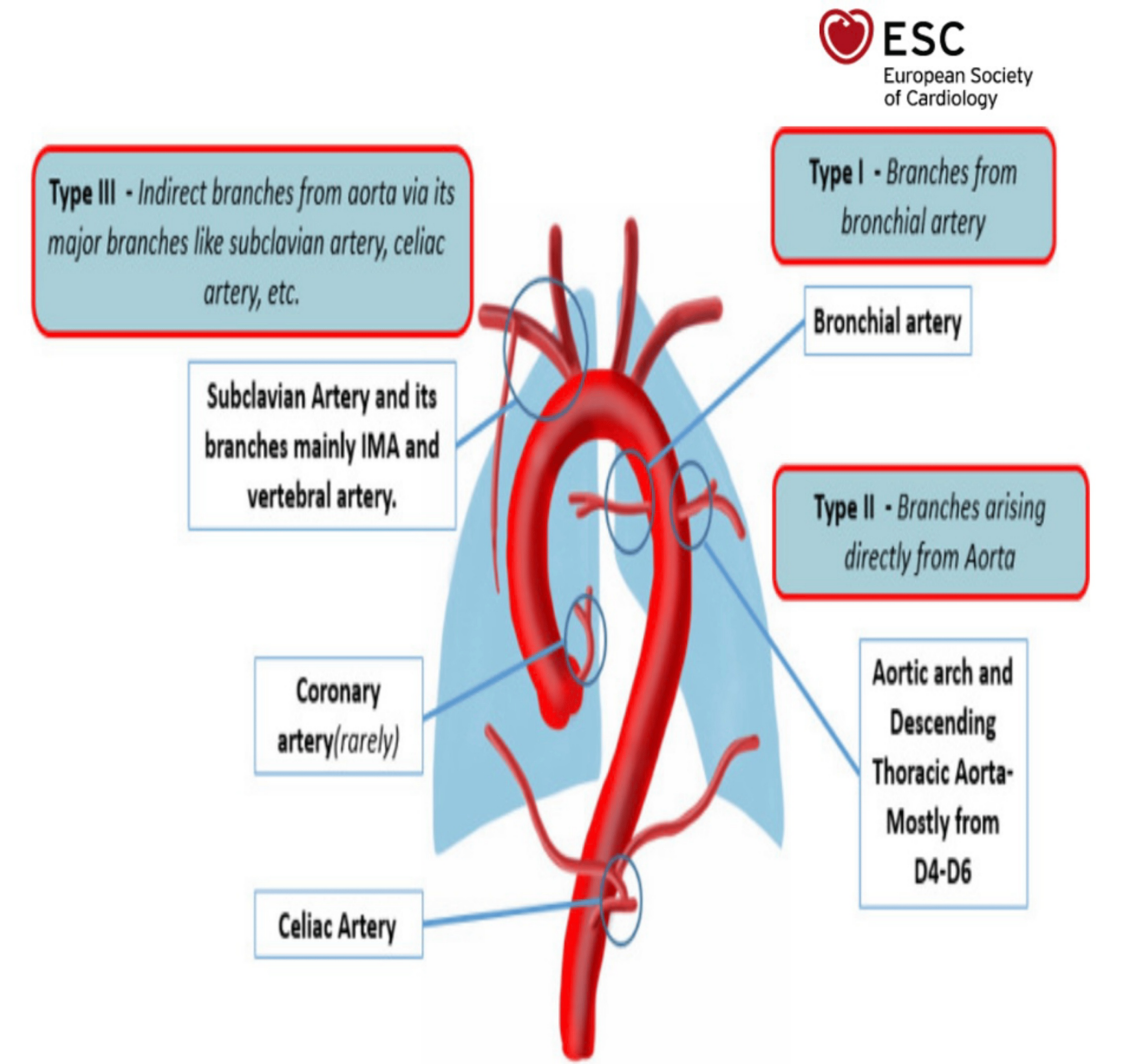
MAPACs pictorial depictions as well as anatomical classification. Our patient’s subtype was undocumented as she refused to consent to undergoing scans to characterize her subtype MAPACs: multiple aortopulmonary connections

It was emphasized that, due to her bidirectional shunt and multiple aortopulmonary connections (MAPACs), a certain degree of desaturation was expected based on the pathophysiology explained earlier (Figure 7). The chest physiotherapy team suggested breathing exercises, contributing to her recovery. Furthermore, according to records, she has had a right heart catheterization in recent years, which confirmed precapillary pulmonary hypertension with an irreversible phenomenon.

Follow-up and outcome

Our patient showed remarkable improvement after starting the new medications. She maintained oxygen saturations between 83% and 86% on room air without excessive breathlessness. Upon discharge, a one-week follow-up and a subsequent four-month appointment with the ACHD and heart failure teams were scheduled for ongoing care. She remained clinically stable at several months of follow-up.

**Figure 8:**
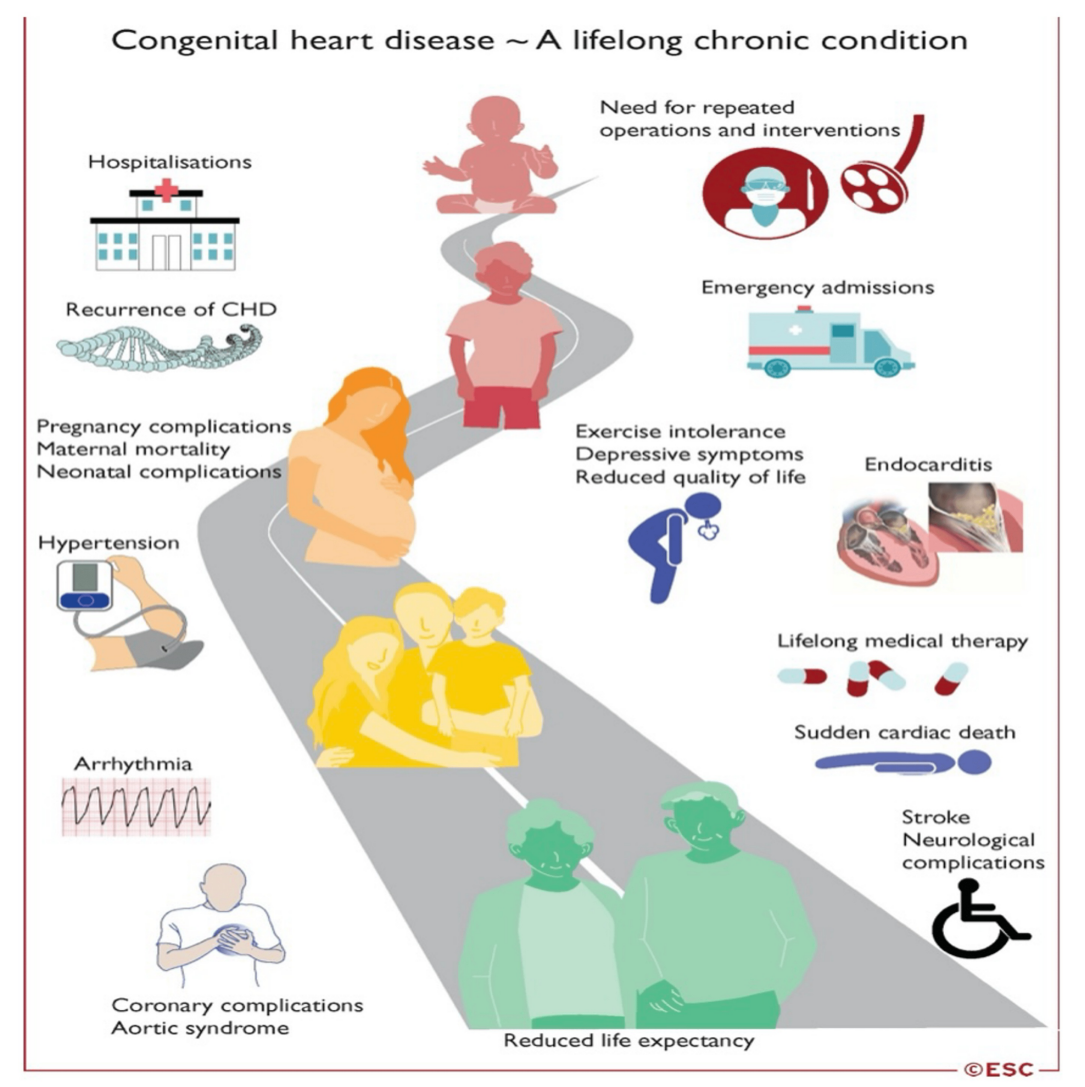
Image with life road of congenital heart disease (CHD) ESC: European Society of Cardiology Photo credit ESC

CHD is a lifelong chronic condition (Figure 8). Her long-term plan from the congenital team was for consideration of a heart-lung transplant. This will be discussed further in the cardiothoracic clinic and multidisciplinary meeting and communicated to the patient. A palliative or conservative approach would be considered in the event of clinical deterioration in the future, as she is deemed inappropriate for major surgical intervention. 

## Discussion

ES results from an untreated congenital cardiac defect causing intracardiac communication, leading to pulmonary hypertension, flow reversal, and cyanosis. The central pathophysiology of ES is pulmonary hypertension caused by long-standing left-to-right shunt, which eventually leads to vascular remodeling of the pulmonary arteries [3,4]. This increases pulmonary resistance, causing the right ventricular pressure to exceed left ventricular pressure, resulting in right-to-left shunting. This altered shunt leads to deoxygenated blood bypassing the lungs, causing chronic hypoxemia and low baseline oxygen saturation. The overall impact is cyanosis, fatigue, and other complications like polycythemia and right heart failure.

ES was first described in 1897, a patient with dyspnoea and cyanosis since infancy developed heart failure and fatal massive haemoptysis, revealing a large VSD and overriding aorta during autopsy [4,5]. This marked the initial recognition of a link between a major congenital cardiac shunt defect and pulmonary hypertension onset [6,7].

In the initial seven years, increased pulmonary blood flow via a left-to-right shunt leads to microvascular changes influenced by altered biological hormone levels such as endothelin-1, thromboxane, platelet activation, and vascular endothelial growth factors. 

Pulmonary hypertension (PH) is defined as mean pulmonary arterial pressure (mPAP) >20 mmHg at rest, with group 1 PH (left-sided heart disease, as in our case) defined by pulmonary arterial wedge pressure (PAWP) >15 mmHg [8,9,10]. According to the European guidelines, the therapeutic goal in this cohort of patients is to reduce pulmonary vascular resistance, improve symptoms, enhance functional class and exercise capacity, and ultimately improve survival outcomes [11].

Pulmonary arterial hypertension (PAH)-targeted pharmacotherapy is therefore the cornerstone of modern Eisenmenger management. Among available agents, endothelin receptor antagonists (ERAs) have the strongest evidence. The BREATHE-5 trial demonstrated that bosentan significantly improved six-minute walk distance (6MWD), pulmonary vascular resistance (PVR), mean pulmonary artery pressure (mPAP), and functional class in Eisenmenger patients [12]. Macitentan, another dual ERA, was evaluated in the MAESTRO trial, showing benefits in NT-proBNP and PVR but failing to achieve statistical significance in 6MWD in a heterogeneous cohort of Eisenmenger patients [13]. Nonetheless, the broader SERAPHIN trial confirmed that macitentan reduces morbidity and mortality in PAH populations [14].

Other pharmacologic strategies include phosphodiesterase-5 inhibitors (PDE-5i) such as sildenafil and tadalafil, which observational and smaller prospective studies have shown to improve functional capacity and haemodynamics in ES [15]. For patients who remain symptomatic despite oral therapy, escalation to prostacyclin or prostanoid therapies (inhaled, subcutaneous, or intravenous) is considered. However, these agents present challenges in Eisenmenger patients, including the risk of worsening right-to-left shunt, venous access issues, and higher infection risk [16].

Combination therapy is increasingly adopted, typically through sequential introduction of drug classes when monotherapy fails. While the evidence in ES is less robust than in idiopathic PAH, emerging data suggest incremental benefits [16]. More recently, novel and emerging therapies such as sotatercept, which targets the BMP/TGF-β pathway, have shown promise in PAH, including in patients with repaired CHD [16].

Historically, Eisenmenger patients faced early mortality, with reported survival rates of 77% at 15 years and 42% at 25 years [17,18]. Common causes of death include heart failure, sudden arrhythmia, or pulmonary haemorrhage. Poor prognostic markers include chronic hypoxemia, limited exercise tolerance, polycythaemia, and severe pulmonary hypertension. Our patient’s relatively preserved daily functioning despite chronic desaturation illustrates how adaptive mechanisms may modulate the clinical course, similar to patients with chronic cardiorespiratory disorders such as COPD.

Management of ES, therefore, requires proactive, multidisciplinary care to minimize complications such as paradoxical embolic stroke, pulmonary haemorrhage, right heart failure, and arrhythmias [19]. While corrective surgery is generally contraindicated once irreversible pulmonary vascular changes have developed, select advanced cases may be considered for lung or heart-lung transplantation [20]. Supportive care, including anticoagulation, infection vigilance, cautious use of vasodilators, and avoidance of pregnancy, remains central to optimizing quality of life and prognosis.

## Conclusions

In conclusion, this case underscores the critical importance of maintaining a high index of suspicion for CHD, particularly ES, in patients presenting with decompensated heart failure and atypical findings such as chronic hypoxaemia and polycythemia. Timely recognition of such underlying conditions is essential to differentiate them from other pathologies, ensuring accurate diagnosis and preventing inappropriate or delayed treatment strategies. Clinicians should remain vigilant for subtle signs that may suggest a prolonged, undiagnosed cardiac anomaly, particularly when conventional explanations for heart failure symptoms are insufficient.

Furthermore, this case highlights the pivotal role of echocardiographic evaluation in identifying structural abnormalities like a large VSD and features consistent with Eisenmenger physiology. It also emphasizes the necessity of revisiting the classification and management strategies for pulmonary hypertension in the context of congenital heart disease. With advancements in pharmacologic therapies and heart failure management, there is growing potential to improve symptom control and quality of life in this patient population. Ultimately, in the group of patients where there are irreversible intracardiac haemodynamic changes, palliative care is often the final resort, particularly in low-resource environments. 
